# Determination of 3- and 4-chloromethcathinone interactions with plasma proteins: study involving analytical and theoretical methods

**DOI:** 10.1007/s11419-023-00677-7

**Published:** 2023-12-18

**Authors:** Piotr Holowinski, Michal P. Dybowski

**Affiliations:** Faculty of Chemistry, Department of Chromatography, Institute of Chemical Sciences, Maria Curie Sklodowska University in Lublin, 20-031 Lublin, Poland

**Keywords:** Chloromethcathinone, Drug–protein binding, Protein-binding degree determination, NMR ligand observed techniques, Molecular docking

## Abstract

**Purpose:**

The purpose of this paper was to determine 3- and 4-chloromethcathinone (3- and 4-CMC) binding degree and possible binding interaction modes with human serum albumin (HSA) using analytical and theoretical methods.

**Methods:**

Experimental determination of 3- and 4-CMC binding degree with HSA was performed using gas chromatography–tandem mass spectrometry preceded by the equilibrium dialysis (ED) and ultrafiltration (UF). Nuclear magnetic resonance (NMR) spectroscopy was used to determine 3- and 4-CMC epitope-binding maps and possible binding sites in HSA. The molecular docking and molecular dynamics were employed to obtain detailed information about binding modes of 3- and 4-CMC enantiomers in HSA.

**Results:**

As follows from the presented data, the degree of binding of 3- and 4-CMC is at a similar level of approx. 80%. This indicates a relatively strong binding of CMC to plasma proteins. The model studies employing the NMR spectroscopy and molecular simulations indicate that both CMCs bind to HSA. The whole 3- and 4-CMC molecules are embedded in the binding sites, with aromatic moieties being in the closest contact with the HSA residues. Moreover, conducted experiments show that  Sudlow site II is the main binding center for 3- and 4-CMC and  Sudlow site I acts as the secondary binding site.

**Conclusions:**

Although many studies describe pharmacological and toxicological properties of synthetic cathinones (SC), the data taking SCs binding in plasma into consideration are scarce. To our knowledge, this is the first report presenting comprehensive experimental and theoretical characterization of 3- and 4-CMC binding with plasma proteins.

**Supplementary Information:**

The online version contains supplementary material available at 10.1007/s11419-023-00677-7.

## Introduction

According to the European Monitoring Centre for Drugs and Drug Addiction (EMCDDA) Council Framework Decisions, new psychoactive substances (NPSs) are one of the most rapidly developing groups of illicit drugs [1, 2]. Synthetic cathinones (SCs) are the second largest group of NPSs monitored by the EMCDDA since they were identified in 2005 [3]. Numerous SCs with wide range of structural features have appeared throughout years; however, chlorine-containing cathinones such as 3- and 4-chloromethcathinone (3- and 4-CMC), which first appeared in 2000s, are still prevalent in the illicit drug market [4].

SCs elicit their pharmacological effects by elevating extracellular levels of monoamine neurotransmitters. A chemical structure of the SCs determines their affinity and selectivity for particular monoamine transporting proteins, dopamine transporter (DAT), serotonin transporter (SERT), and noradrenaline transporter (NET), and whether they are monoamine reuptake inhibitors or monoamine releasers [1].

After being distributed in circulating blood, drugs, including SCs, bind to plasma proteins in varying degrees. In general, such binding is reversible and an equilibrium exists between bound and free molecular species. It is commonly stated unless there is a specific transport system, only free drug molecules are able to cross membrane barriers and are distributed to tissues. Hence, only the free drug fraction (free form) is able to exert pharmacological and toxicological effects [5–8].

There are many components in plasma that are capable to bind drugs, however, two plasma proteins, human serum albumin (HSA) and α_1_ acid glycoprotein (AGP), are present in relatively high and moderate quantities, respectively, and are able to bind a variety of drugs with sufficient affinity to have a significant effect on drug disposition and action [9].

Many methods have been proposed to assess protein–drug binding based on diverse analytical tools. They can be divided into separative and non-separative methods. The separation methods include the following: (1) size-exclusion chromatography [10, 11] and affinity chromatography [12, 13]; (2) capillary electrophoretic techniques [14, 15]; and (3) equilibrium techniques which involve the application of equilibrium dialysis (ED) [16, 17], ultrafiltration (UF) [18, 19] and ultracentrifugation (UC) [5, 20, 21]. The latter group can be considered as the most important class of separative techniques as the equilibration methods provides a relatively high accuracy, simplicity of implementation and very low cost of research equipment.

Currently, much attention is paid to non-separative methods, which include (1) calorimetric techniques involving isothermal titration calorimetry (ITC) [22, 23] and differential scanning calorimetry (DSC) [24, 25]; (2) surface plasmon resonance-based assays (SPR-based techniques) [26, 27], and (3) spectroscopic techniques such as ultraviolet–visible (UV–VIS) spectroscopy [28, 29], fluorescence spectroscopy [30, 31], infra-red (IR) spectroscopy [32, 33], nuclear magnetic resonance (NMR) spectroscopy [34, 35], optical rotatory dispersion (ORD) spectroscopy [5, 36], and circular dichromism (CD) measurement [37, 38]. NMR spectroscopy is characterized by the greatest possibilities in relation to the other non-separative methods. Saturation transfer difference (STD) measurement [39–41] and Water-Ligand Observed via Gradient Spectroscopy (WaterLOGSY) [39, 41, 42], being the most common NMR techniques for the protein–drug-binding characterization, allow not only to determine whether a drug interacts with protein, but also to make the use of these methods possible to determine epitope-binding maps and drug-binding sites.

In addition to the separative and non-separative techniques, the molecular simulation methods are commonly applied for characterization of the drug–protein binding. Molecular docking [43, 44] and molecular dynamics [45, 46] are typically employed. These simulation techniques are applied to study binding phenomena with atomic resolution, i.e., they allow to determine interactions of drug functional groups with particular protein residues. In the case of molecular dynamics, time evolution of drug–protein interaction is examined as well.

Although many studies describe pharmacological and toxicological properties of synthetic cathinones, the data considering SCs binding in plasma are scarce. Binding degrees of SCs with plasma proteins were determined only for exemplary cathinones resulting in binding degrees in range from 59 to 77% in human plasma [47]. Considering the drug binding with plasma proteins, the determination of the nature of protein–drug interactions, i.e., drug-binding sites in protein, interactions between drug groups and protein residues, thermodynamic parameters and influence of binding on the structure of a protein, is also pivotal for the understanding of binding phenomena. In case of SCs, the mephedrone binding by bovine serum albumin (BSA) was studied with the use of the spectroscopic techniques (UV, fluorescence and IR spectroscopy techniques) and molecular docking leading mainly to the thermodynamic data describing binding events and brief description of changes in BSA secondary structure [48]. Hence, the comprehensive research is still needed to explore the most important features of SCs interactions with plasma proteins. Thus, the objective of this paper is to examine plasma protein binding of (3- and 4-CMC), which we chose as model SCs for separative, NMR spectroscopy and molecular simulations experiments. In this study, it was decided to:

- determine the 3- and 4-CMC protein-binding degree using the separative methods such as ED and UF
- employ NMR techniques to examine 3- and 4-CMC interactions with HSA and to determine epitope-binding maps and possible binding sites of these SCs in HSA
- determine binding modes of 3- and 4-CMC enantiomers in binding sites of HSA using the molecular docking and molecular dynamics
- compare the results obtained from separative methods, non-separative methods and molecular simulations.

## Experimental

### Materials

Methanol (LC/MS grade) was purchased from Merck (Warsaw, Poland). Phosphate-buffered saline (PBS), HSA (97%), warfarin (analytical standard), diazepam (analytical standard), deuterium oxide (D_2_O), DMSO-d_6_ were acquired from Sigma-Aldrich (Poznan, Poland). Deionized water was prepared by the Milli-Q system (Millipore Sigma, Bedford, MA, USA).

3- and 4-CMC standards were donated by the forensic laboratory of the Provincial Police Department in Lublin. Working solutions were stored in stable conditions at – 20 °C.

The blood/plasma samples were collected by a registered nurse from volunteers, after obtaining their informed consent, using a single closed system containing an S-Monovette coagulation activator, according to the manufacturer instructions (Sarstedt AG, Nümbrecht, Germany), and thoroughly mixed in order to maintain their homogeneity.

### Gas chromatography–tandem mass spectrometry (GC–MS/MS) measurements

Qualitative and quantitative analyses of CMC were conducted using a gas chromatograph hyphenated with a triple quadruple tandem mass spectrometer detector (GCMS-TQ8040; Shimadzu, Kyoto, Japan) equipped with a ZB5-MSi fused-silica capillary column (30 m × 0.25 mm i.d., 0.25 µm film thickness; Phenomenex, Torrance, CA, USA). Helium (grade 5.0) as carrier gas and argon (grade 5.0) as collision gas were used. Column flow was 1.5 mL/min, and 1 µL of the sample was injected by an AOC-20i + s type autosampler (Shimadzu). The injector was working in high pressure mode (250.0 kPa for 1.5 min; column flow at initial temperature was 4.90 mL/min) at the temperature of 310 °C; the ion source temperature was 185 °C.

For qualitative purposes, the full scan mode with range 40–550 m*/z* was employed, and for quantitative analyses, the multiple reaction monitoring (MRM) mode was used. Three MRM transitions of the highest intensity were selected for further experiments: 197 > 139 (collision energy (CE) = 15 eV), 197 > 58 (CE = 15 eV) and 139 > 111 (CE = 15 eV) for 3- and 4-CMC.

### Experimental drug-free form determination

#### Followed by equilibrium dialysis (ED)

The equilibrium dialysis was performed using two-chambered plexiglass dialysis cells each of 1 mL capacity (Technilab Instrument Corp., Pequannock, N.J.). The cells were separated by a thick membrane with a molecular weight cutoff of 6000–8000 from Thermo Fisher Scientific (USA, Hampton, New Hampshire). The test solutions, prepared in human serum and PBS, were placed in one compartment, called the retentate, and PBS was placed in the other compartment, called the permeate. The cells were equilibrated at room temperature for 16 h before the retentate and permeate solutions were removed (sufficient time to reach equilibrium). The extracted samples were centrifuged at 2000 x g for 4 min and the supernatant were transferred for the GC–MS analysis. The 3- and 4-CMC binding degrees (*F*_*u*_*%*) were determined according to [49]:

$${F}_{u}\%=\frac{{D}_{{\text{t}}}-{D}_{{\text{f}}}}{{D}_{{\text{t}}}}100\%$$

where *D*_t_ is the total compound concentration in the plasma compartment and *D*_f_ is the concentration of the compound in free form in the PBS compartment.

#### Followed by ultrafiltration (UF)

Protein-binding measurements of 3-CMC and 4-CMC were conducted using the ultrafiltration technique performed on Millipore Amicon MPS (USA, Burlington, Massachusetts) units. YM-10 membranes (product no. 40424, Millipore, Bedford, MA, USA) of 10 kDa molecular mass cutoff were used in this experiment. The units were centrifuged in a thermostated centrifuge (MPW-350-RH, MPW Med. Instruments, Poland) at the temperature of the human body (37 °C). 3- and 4-CMC solutions (20 µg/mL) were prepared in HSA and PBS and 1 mL was added to the pretreated filter cups of ultrafiltration units and were equilibrated for 1 h at room temperature. After the attachment of an ultrafiltrate collection container, the unit was centrifuged at 1100 x g till 400 µL of ultrafiltrate was obtained (usually for about 15–20 min). Two hundred µL of the filtrate samples from the bottom reservoir was diluted in 800 µL of methanol and transferred for the analysis. In addition, a calibration curve was prepared in PBS. The samples and calibrators were combined with the equivalent volume of methanol before the analysis.

### NMR spectroscopy measurements

NMR spectroscopy measurements were conducted at 298 K using the Bruker (USA, Billerica, Massachusetts) Ascend 600 MHz instrument equipped with 5 mm TXI probe. PBS in D_2_O (pD 7.4) and PBS in H_2_O (pH 7.4) were prepared. There were prepared the following stock solutions: 3-CMC in D_2_O (40 mM), 4-CMC in D_2_O (40 mM), HSA in D_2_O PBS (0.2 mM), HSA in H_2_O PBS (0.2 mM), Warfarin in DMSO-d_6_ (40 mM) and Diazepam in DMSO-d_6_ (40 mM). The samples for STD and WaterLOGSY measurements were prepared in 500 µL of D_2_O PBS and in 500 µL of H_2_O:D_2_O 90:10 PBS. The samples for binding studies contained 20 µM HSA and 800 µM 3-CMC or 800 µM 4-CMC. The solutions used for the competition studies additionally contained 800 µM of binding site probe. Warfarin and diazepam were used as binding site probes for  Sudlow site I and  Sudlow site II, respectively. The prepared samples contained the same amounts of DMSO-d_6_. The blank samples without HSA were prepared with the same concentrations of ligands and binding site probes.

The STD pulse sequence employed the selective saturation of protein resonances with the series of 50 ms soft Gaussian shaped pulses. Following saturation times were employed: 0.5 s, 1 s, 1.5 s, 2 s, 3 s, 4 s, 5 s, and 6 s. The irradiation in the positions 0.0 ppm and 40 ppm was performed for the on-resonance saturation and off-resonance saturation, respectively. The STD pulse sequence also includes 30 ms spinlock filter and excitation sculpting for water suppression. The STD spectra were obtained with 512 scans, the spectral width 12 ppm.

The epitope-binding maps were determined according to the Mayer et al. [50]. The STD amplification factors were defined as

$${\eta }_{i}=\frac{{I}_{0}-{I}_{{\text{STD}}}}{{I}_{0}}\varepsilon$$

where *η*_*i*_ is the STD amplification factor of nuclei *i*, *I*_0_ and *I*_STD_ are the off-resonance and on-resonance intensities of protons signals, and *ε* is the ligand concentration to protein concentration ratio. The STD build-up curves, i.e., the relations *η*_*i*_ = *f(t)*, where *t* is the saturation time, were fitted to the function:

$${\eta }_{i}={{\text{STD}}}_{{\text{max}}}\left(1-{\text{exp}}\left(-{k}_{{\text{sat}}}t\right)\right)$$

where STD_max_ is the maximum of STD amplification factor, *k*_sat_ is the saturation rate constant, and t stands for the saturation time. The initial growing rate of amplification factor, denoted as STD_0_, was defined as

$${{\text{STD}}}_{0}=\underset{t\to 0}{{\text{lim}}}\frac{d{\eta }_{i}}{dt}={{\text{STD}}}_{{\text{max}}}{k}_{{\text{sat}}}$$

The obtained STD_0_ values can be considered as the STD signals in the absence of differences in the spin–lattice relaxation as well as spin diffusion of magnetization within the ligand [50]. Values of STD_0_ were normalized with respect to the largest value of STD_0_ obtained for 3-CMC and 4-CMC, respectively. Thus, normalized STD_0_ obtained for the 3-CMC and 4-CMC nuclei was used as a measure of saturation transfer in the epitope-binding maps.

The WaterLOGSY pulse sequence with excitation sculpting for water suppression, 30 ms spin lock and 2.2 ms mixing time for the generation of observed signals was employed. The WaterLOGSY spectra were obtained with 512 scans, the spectral width 12 ppm.

### Molecular docking

AutoDock 4.2 and AutoDockTools [51] were used for the molecular docking studies of 3-CMC and 4-CMC enantiomers in  Sudlow sites of HSA. The structures of HSA were obtained from the Research Collaboratory for Structural Bioinformatics Protein Data Bank, PDB ID: 1H9Z [52] (HSA-warfarin complex) and 2BXF [53] (HSA-diazepam complex) for docking in Site I and Site II, respectively. The starting geometries of (S)-4-CMC, (R)-4-CMC, (S)-3-CMC and (R)-3-CMC were optimized with the MMFF94 force field in Avogadro [54–59]. Gasteiger charges and hydrogen atoms of polar groups were added to the protein and the ligands. Docking of cathinone ligands in HSA was performed with the grid boxes of the size: 52 Å × 74 Å × 50 Å along the X, Y and Z axes, respectively, and the grid spacing 0.375 Å for Site I; 76 Å × 62 Å × 60 Å along the X, Y and Z axes, respectively, and the grid spacing 0.375 Å for Site II. The genetic algorithm was employed to perform docking simulations with 100 runs, the maximum number of 27,000 generations and 2 500 000 energy evaluations. Considering Tyr 411 as a residue being pivotal for the binding of ligands in site II its rotatable bonds were set free to rotate along with the rotatable bonds of cathinone ligands during the docking simulations. The docking poses with the highest binding energies were considered as the optimal binding modes and inspected using LIGPLOT + [60] and PyMOL.

### Molecular dynamics (MD) simulations

MD simulations were conducted using the NAMD 2.14 package (Theoretical Biophysics Group University of Illinois and Beckman Institute, USA, Mathews, Urbana, IL) [61] with CHARMM36 force field [62–64] and CHARMM General Force Field [65]. The optimal docking poses obtained for the HSA complexes with 3-(R)-CMC in  Sudlow sites I and II were used as initial conformations. Simulation setups were generated using  CHARMM-GUI [66–68]. The ligand–HSA complexes were solvated with the TIP3P water molecules in simulation boxes of the dimensions 112 Å × 112 Å × 112 Å along the X, Y and Z axes, respectively. Na^+^ ions were placed in random positions to neutralize simulation system. After the minimization of the obtained ligand–HSA complex, the following simulation steps were performed: 200 ps of thermal equilibration at 300 K in the NVT ensemble, 200 ps of equilibration at 1 atm and 300 K using the NPT ensemble, and 20 ns of production run in the NPT ensemble at 300 K and 1 atm. The Verlet algorithm with 2 fs time step was used for the integration. Constant temperature and pressure scaling were assured with the Langevin dynamics and Langevin piston method, respectively. The particle mesh Ewald method was used to compute long-range electrostatic interactions. The analysis of MD trajectories obtained during production run, i.e., calculation of Root Mean Square Displacement (RMSD) with atoms positions at the beginning of production run being used as a reference, was performed with VMD [69].

## Results

### Determination of 3- and 4-CMC free drug forms

The choice of the appropriate method of free drug isolation is extremely important for reliability of the results obtained with its use. The described experiments were conducted using the two methods of CMC binding degree determination –UF and ED.

When the ED is used as an isolation method for the studies of drugs binding to the plasma albumin, it is recommended to determine the relationship between the fraction of free drug and its total concentration for each drug in the drug–albumin system.

In order to determine the relationship between the fraction of free CMC and its total concentration, it was necessary to determine the time required for this drug to reach equilibrium in the equilibrium dialysis process. The conducted studies indicate that in the case of both 3- and 4-CMC, the concentration equilibrium between the dialyzer compartments is reached after about 16 h. This is not surprising because the CMC molecule has a low molecular weight and is characterized by a high diffusion coefficient.

When working with biological samples, the risk of their degradation by microorganisms must be taken into account. This phenomenon is particularly unfavorable when studying the degree of drug binding due to the fact that microbial organisms can lead to the degradation of binding proteins, and thus to the disturbance of the drug–protein-binding process and, consequently, to obtaining erroneous results. It was, therefore, decided to check after what time, under the established conditions, in the process of equilibrium dialysis, samples containing drug–protein systems begin to degrade. For this purpose, multi-day dialysis of a solution containing 3-CMC and plasma at room temperature (24 °C) was carried out. The obtained data indicate that in the case of the temperature selected for further research (room temperature), changes in the free fraction of CMC take place in the time interval between 68 and 82 h, which is much longer than the time of reaching equilibrium by the system (16 h).

UF is considered as a rapid alternative to ED. This method is similar to ED except that the analysis speed is increased by application of centrifugal force to facilitate the solution transport through the membrane.

**Fig.1:**
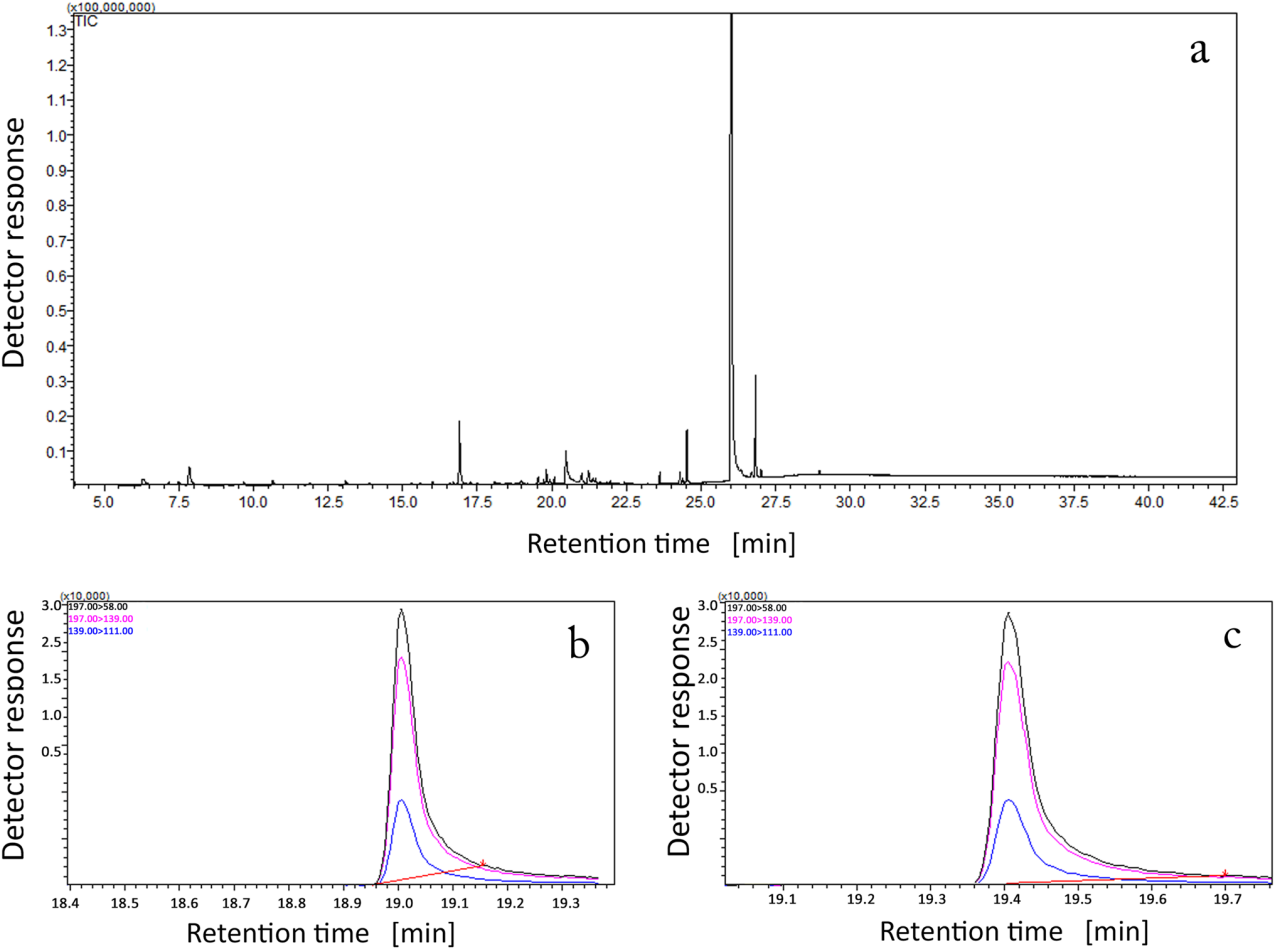
GC–MS/MS chromatograms from UF extracts obtained in the **a** scan mode and MRM mode for **b** 3-CMC and **c** 4-CMC

Figure 1 shows the exemplary GC–MS/MS chromatograms obtained in the (a) scan mode and MRM mode for (b) 3-CMC and (c) 4-CMC, after the UF process. Table 1 presents the experimentally determined degree of 3- and 4-CMC binding using the ED and UF. As the results for the presented data, the degree of binding of both analytes is at a similar level of approx. 80%. This indicates relatively strong binding of the substance to the plasma proteins in relation to other drugs reported in literature, such as tryptophan, thiopental, rifabutin, or mepivacaine (binding degree equals 80%, 80%, 85% and 84%, respectively), which are recognized as drugs strongly binded to plasma proteins [70]. In addition, there is a slight difference in the binding degree values between the used techniques (79.1% and 79.3% for 3- and 4-CMC for ED and 80.2% and 80.5% for 3- and 4-CMC for UF), which is not much surprising as it results from the characteristics of the individual methods.

**Table 1:** Binding degree of 3- and 4-CMC with human plasma proteins estimated using equilibrium dialysis (ED) and ultrafiltration (UF)

Compound	Ultrafiltration	Equilibrium dialysis
	Protein-binding degree [% ± SD] (*n* = 5)	
3-CMC	80.2 ± 0.5	79.1 ± 0.4
4-CMC	80.5 ± 0.6	79.3 ± 0.4

### Model studies with HSA

The NMR spectroscopic methods, i.e., STD and WaterLOGSY were employed to assess whether 3-CMC and 4-CMC bind to HSA and to examine the nature of binding process. Both techniques are based on the nuclear Overhauser effect (NOE) occurring between the ligand nuclei and the protein-binding center nuclei allowing to investigate the interactions between the small ligands and proteins (or other macromolecules) [39]. STD is based on the selective saturation of protein resonances, which is subsequently transferred to the ligand nuclei being in the close contact with the protein nuclei [40, 41]. It results in the attenuation of ligand resonances. The most significant signal decrease occurs for the nuclei closest to the protein [40, 41]. WaterLOGSY employs irradiation of water to produce NOE for the bound ligands. Water molecules residing in the binding cavity of protein transfer magnetization to the interacting ligands via the multiple pathways [41, 42].

**Fig. 2:**
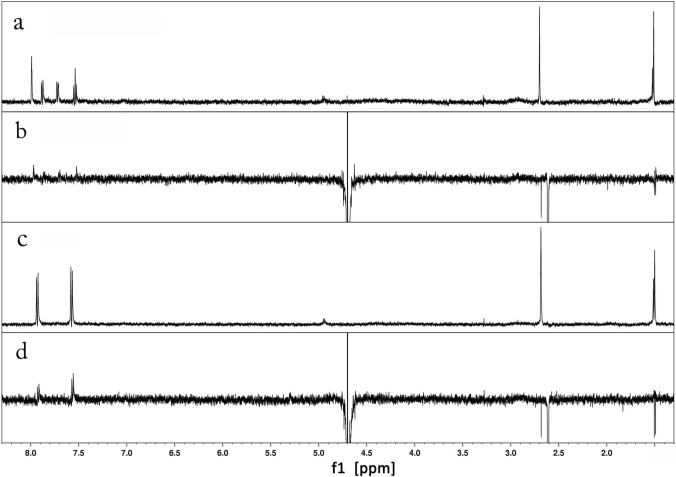
**a** STD NMR and **b** WaterLOGSY NMR spectra of 3-CMC (800 µM) with HSA(20 µM) and **c** STD NMR and **d** WaterLOGSY NMR spectra of 4-CMC (800 µM) with HSA (20 µM)

Figures 2a–d shows the STD and WaterLOGSY spectra of 3-CMC in the presence of HSA (Fig. 2a and b) as well as those of 4-CMC in the presence of HSA (Fig. 2c and d), where Fig. S1a–b (see supplementary materials) shows the ^1^H NMR spectra for 3- and 4-CMC obtained using the water suppression technique employing analogous suppression conditions as in the acquisition of STD and WaterLOGSY spectra. The saturation transfer difference spectra of 3-CMC and 4-CMC contain significant signals of aromatic protons and methyl protons indicating their close contact with the protein. The WaterLOGSY spectrum of 3-CMC shows the inverted signals of minor intensities from aromatic protons whereas the resonances of aliphatic protons are only attenuated without their inversion. Similar signals can be found in WaterLOGSY of 4-CMC with the aromatic protons signals showing greater intensity compared to the analogues signals of 3-CMC. The STD and WaterLOGSY studies results indicate clearly that both isomers of chloromethcathinone bind to HSA.

**Fig. 3:**
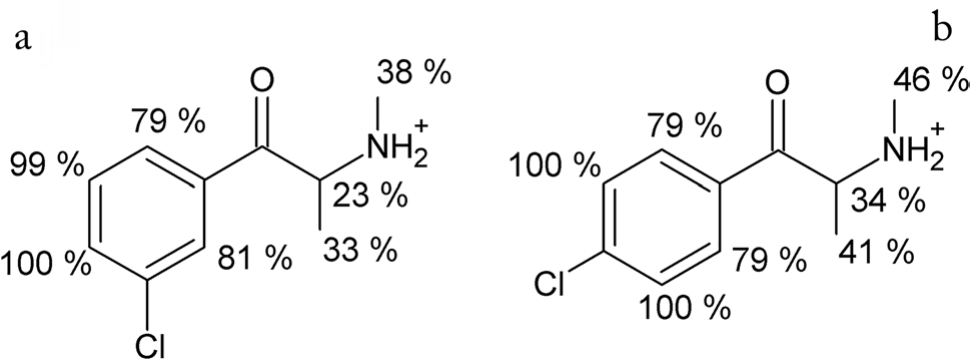
Epitope mapping of **a** 3-CMC and **b** 4-CMC interactions with HSA. Normalized values of STD_0_ for observed protons of 3- and 4-CMC are presented, with highest STD_0_ observed for 3- or 4-CMC as a 100% and as a ratio of STD_0_ and maximum of STD_0_ for the remaining protons

Identification of ligand groups interacting with the protein residues is crucial for understanding the ligand–protein interactions. Thus, the epitope-binding maps were created using STD for the quantitative assessment of the contribution of particular groups of 3-CMC and 4-CMC in the binding process with HSA. The most significant STD signals occur for the ligand nuclei closest to the protein as the STD experiment is based on the saturation transfer between the ligand and protein via NOE. The initial growing rates of STD signals, denoted as STD_0_, were employed to determine the epitope-binding maps for 3-CMC and 4-CMC. The obtained fitting parameters and the STD_0_ values are summarized in Table S1 (see supplementary materials). The values of STD_0_ are presented in normalized scale, in which the highest value of an absolute STD_0_ was considered as a 100% and values of normalized STD_0_ of other nuclei of 3- or 4-CMC were determined with respect to the highest value of absolute STD_0_. As the STD experiment is based on the NOE occurring between drug and protein residues, the values of normalized STD_0_ have following interpretation: the higher the value of normalized STD_0_, the closer the contact of a considered group of 3- or 4-CMC with protein residues and the more significant the interaction of a considered group of 3- or 4-CMC with protein residues. Figure 3a and b shows the epitope-binding maps obtained for 3-CMC and 4-CMC, respectively. The presented normalized STD_0_ values indicate that the aromatic rings act as main binding moieties. The aromatic protons, which are influenced by the electron withdrawing character of the carbonyl group to a lesser extent, give the greatest normalized STD_0_ in the presented epitope-binding maps. This indicates that these protons have the closest contact with the HSA residues. Lower responses from the remaining aromatic protons are observed. Results acquired for the aliphatic groups exhibit much smaller values of the normalized STD_0_ than STD_0_ of aromatic protons indicating that aromatic ring is a main binding group for CMC binding with HSA. This is more pronounced for 3-CMC, whose methyl groups correspond to the normalized STD_0_ equal to 38% and 33% compared to the values obtained for 4-CMC, i.e., 46% and 41%.

HSA is known to contain two specific binding sites, namely  Sudlow site I located in subdomain IIA and  Sudlow site II located in subdomain IIIA [71]. Competitive binding studies of the examined ligand with the known binders of  Sudlow sites are needed to determine the ligand-binding site. Warfarin and diazepam were used as binding site probes for  Sudlow site I and site II, respectively to determine the binding sites of the 3-CMC and 4-CMC. Table 2 shows the relative STD signals intensities for considered CMCs in the presence of 800 µM of binding site probe. It can be observed that both binding probes cause a decrease of STD signals of 3-CMC and 4-CMC indicating that CMCs are bound by  Sudlow site I and site II. The comparison of the STD signals attenuation induced by the presence of binding probes allows to define a main binding site. Higher levels of signals attenuation are observed in the presence of diazepam, thus indicating that  Sudlow site II is a main binding site of 3- and 4-CMC, whereas  Sudlow site I acts as a secondary binding cavity.

**Table 2:** Attenuation of STD_0_ values in the presence of binding site probes for  Sudlow sites I and II of HSA (*n* = 3)

^1^H δ [ppm]	STD_0 with probe_ /STD_0_	
	Diazepam	Warfarin
3-CMC		
7.99	37%	82%
7.87	30%	–^a^
7.71	38%	77%
7.53	–^a^	–^a^
4.90	39%	73%
2.69	37%	69%
1.50	40%	76%
4-CMC		
7.93	43%	60%
7.57	38%	58%
4.96	45%	62%
2.69	49%	61%
1.51	42%	58%

^a^STD_0_ cannot be determined due to the signal overlap with binding probe resonances

### Molecular simulations of CMC-HSA systems

**Fig. 4:**
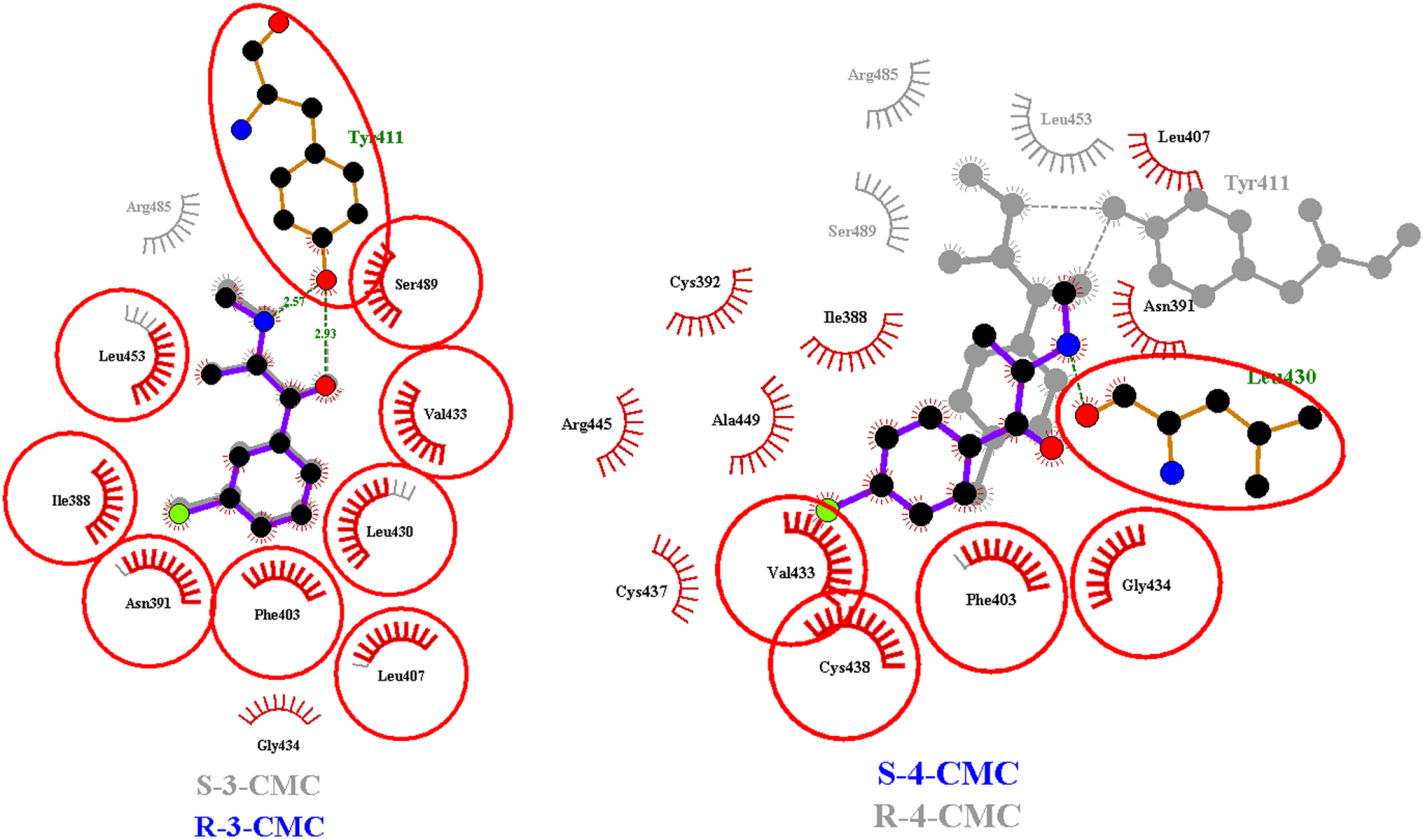
Superimposed Ligplot + diagrams of 3-CMC enantiomers and 4-CMC enantiomers binding modes in HSA  Sudlow site II. Hydrogen bonds are represented by dashed lines and non-bonded interactions are represented by spoked arcs. HSA residues interacting with both 3- or 4-CMC enantiomers are highlighted with red circles and ellipses

**Fig. 5:**
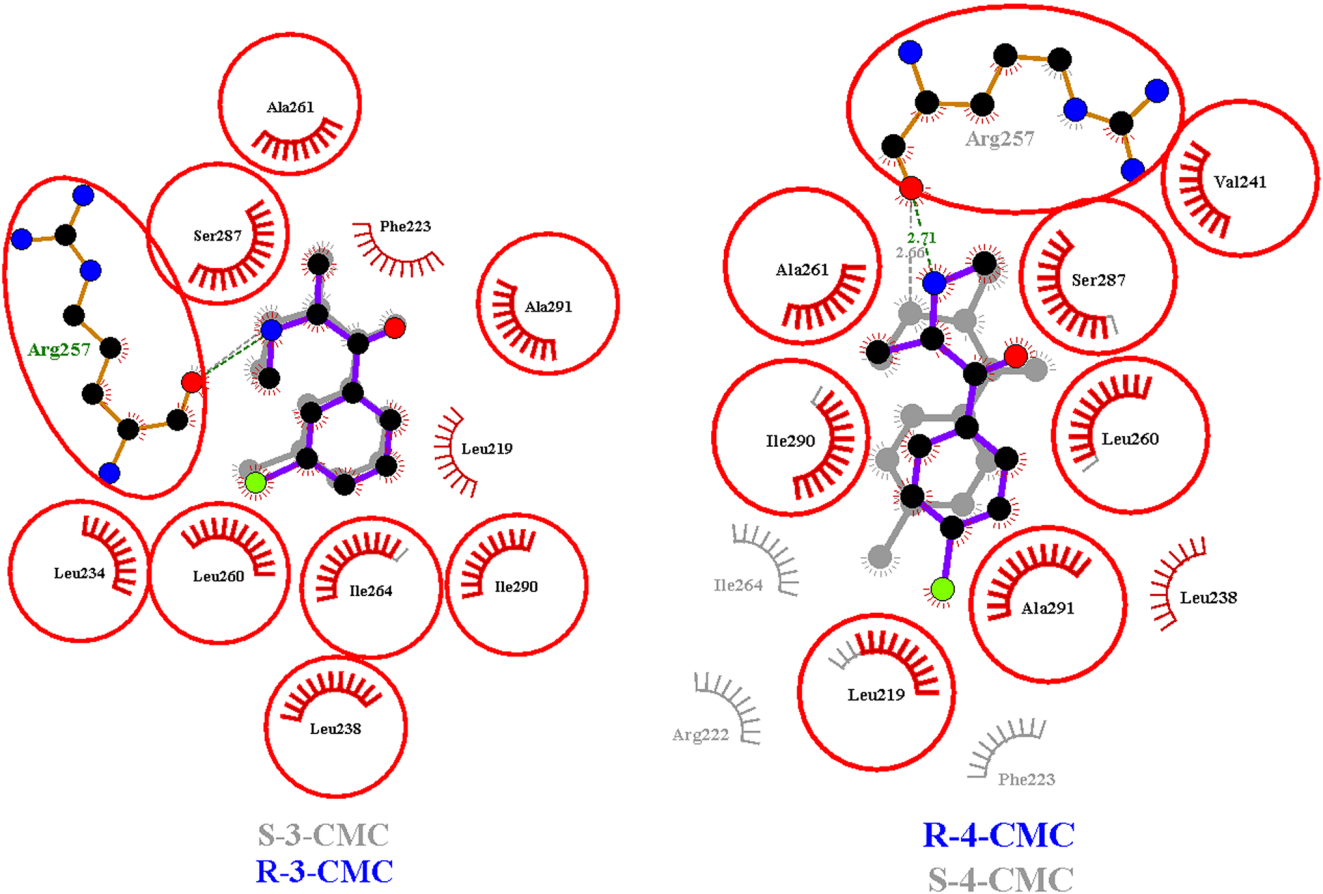
Superimposed Ligplot + diagrams of 3-CMC enantiomers and 4-CMC enantiomers binding modes in HSA  Sudlow site I. Hydrogen bonds are represented by dashed lines and non-bonded interactions are represented by spoked arcs. HSA residues interacting with both 3- or 4-CMC enantiomers are highlighted with red circles and ellipses

Molecular docking and molecular dynamic simulations were employed to investigate the nature of CMCs binding in  Sudlow sites I and II of HSA. The R and S enantiomers of 3-CMC and 4-CMC were investigated to determine whether chirality influences the binding process. According to the literature data [72], SCs exist in the ionic form under the physiological conditions, thus the cationic molecules of 3-CMC and 4-CMC were examined during the simulations. Figures 4, 5 and Figures S2–S3 (see supplementary materials) present best docking poses obtained for R and S CMCs in Sites II and I.

In the case of 3-CMC docking in Site II, i.e., the main binding site of the considered CMCs, both enantiomers interact with numerous hydrophobic residues (Val 433, Leu 430, Leu 407, Phe 403, Gly 434, Ile 388 and Leu 453) and a fewer number of hydrophilic residues: Asn 391, Ser 489 and Tyr 411. Thus, the hydrophobic interactions can be considered as a major force participating in stabilization of the obtained docking poses. The additional stabilization of 3-CMC-HSA complexes results from the formation of hydrogen bonds between Tyr 411 and ketone as well as amine moieties of R- and S-3-CMC. Comparing the docking poses of R and S enantiomers it can be observed that S-3-CMC additionally interacts with the CH_2_ groups of Arg 485 and has a closer contact with Ser 489. In the case of R-4-CMC its optimal docking pose resembles the orientations generated for the enantiomers of 3-CMC with similar hydrophobic interactions, i.e., interactions with Val 433, Phe 403, Gly 434, Leu 430, Leu 453 and with aliphatic parts of Ser 489, Arg 485, and hydrogen bonds formed with Tyr 411. As opposed to the above results, the S-4-CMC docking pose differs significantly from those of remaining isomers. As can be seen in Fig. S2d (see supplementary materials), S-4-CMC is embedded into the further part of subdomain IIIA cavity. Only the amine group of S-4-CMC is involved in the hydrogen binding. Docking of the examined CMCs in Sudlow Site I resulted in similar docking poses for all isomers. The lowest docking poses are stabilized by the dispersive interactions and the hydrogen bond between the carbonyl group of Arg 257 and the amine groups of CMC isomers. The docking poses of CMC enantiomers in Site I differ only in the number of residues interacting via the dispersive interactions with the most significant changes occurring for the S-4-CMC, where additional interactions with Ile 264, Phe 223 and with CH_2_ of Arg 222 are observed and the interactions with Leu 238 do not occur.

Table S2 (see supplementary materials) summarizes the binding parameters from the molecular docking simulations. The binding energies observed in the range from – 5.07 to – 6.67 kcal mol^−1^ and the corresponding dissociation constants are consistent with the typical values obtained for binding between HSA and small organic ligands [73, 74]. The binding parameters show that docking poses obtained for Site II are energetically favored over those acquired for Site I. The changes in the docking energies between isomers in particular binding site can also be observed. The largest binding energies are observed for R-3-CMC and S-3-CMC in the case of site II and site I docking poses, respectively. The influence of chirality on the docking parameters is more pronounced for the enantiomers pairs of 3-CMC in both  Sudlow sites whereas the docking parameters of 4-CMC enantiomers differ to a lesser extent.

**Fig.6:**
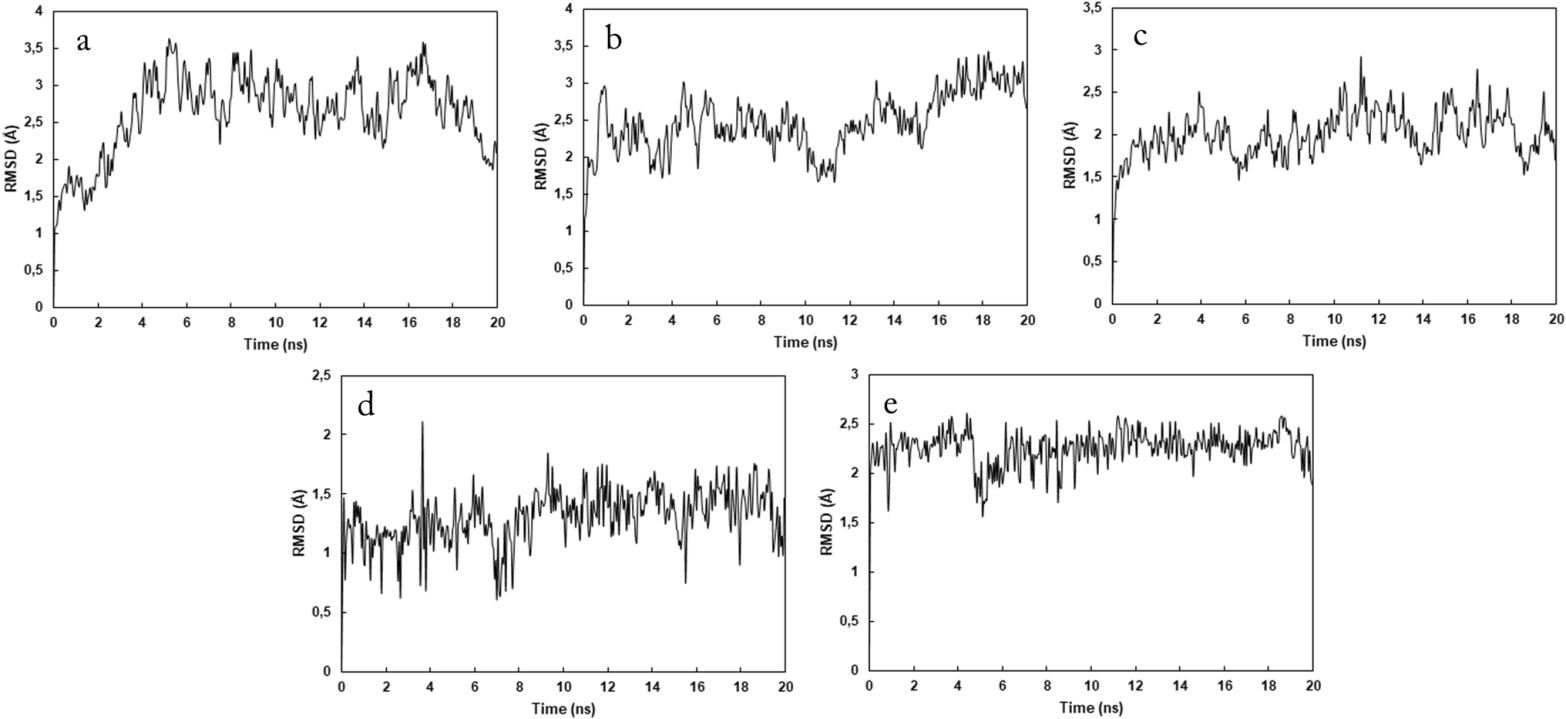
Alpha carbon RMSD of HSA with R-3-CMC embedded into **a** Sudlow site II, **b** Sudlow site I and **c** of apo HSA, and RMSD of R-3-CMC within **d**  Sudlow site II and **e**  Sudlow site I obtained from MD simulations

Molecular dynamics was employed for further evaluation of CMC-HSA interactions. R-3-CMC was chosen as a model isomer for this simulation and the time evolution of its docking poses in the  Sudlow sites was examined (see Experimental section for a detailed description of a employed simulation setup and trajectories analysis). Figure 6a–c present the RMSD of HSA alpha carbons obtained during the simulations of R-3-CMC in  Sudlow Sites and for the free HSA. It can be seen that RMSD reached stable values during the production run, which allows for the analysis of the obtained trajectories. RMSD of R-3-CMC in Sites I and II (Fig. 6d, e) are relatively small, i.e., do not exceed 3 Å which indicates that a ligand molecule exists inside the binding site along the whole simulation and the generated conformations of ligand are close to the initial one. This shows that hydrophobic interactions are prevalent for the stabilization of CMC-HSA complexes during molecular simulations, as the most of the residues existing inside  Sudlow Sites are hydrophobic. Thus, further analysis of MD trajectories was focused on the specific interactions like hydrogen bonding.

The snapshot from the MD simulation for the complex of R-3-CMC in Site II is shown in Fig. S4a (see supplementary materials). The analysis of the trajectories allows to observe that Asn 391 and Tyr 411 are involved in the formation of hydrogen bonds with R-3-CMC which can be illustrated by Fig. S5 (see supplementary materials) showing the time evolution of the distances between R-3-CMC and the HSA residues, in which distances close to and smaller than 3 Å can be correlated with the formation of hydrogen bonds. Hydrogen bonds from the initial docking pose, i.e., the bond of Tyr 411 with the carbonyl and amine moieties are present to the lesser extent, whereas the interactions of CMC amine with carbonyl of Asn 391 last for the longer period of time. In addition, the face-to-edge interactions of CMC aromatic ring with Phe 403 occur (see Fig S4a in supplementary materials).

Figure S4b (see supplementary materials) presents the exemplary snapshot from the trajectory obtained for R-3-CMC in  Sudlow Site I. As opposed to the MD results from Site I, the hydrogen bond with the peptide carbonyl of Arg 257, which was observed in the molecular docking, disappears at the beginning of the simulation. The hydrogen bond with Gln 196 occurring mainly in the 5–10 ns time interval (see Fig. S6 in supplementary materials) is the only specific interaction during the simulation.

## Discussion

The clinical experience, as well as many clinical pharmacologic studies, demonstrates clearly that the measurements of drug concentrations in the plasma correlate far better with the clinical effect than the drug dose does. The ED is still considered as the “gold standard” method for determination of free drug concentration in biological fluids. In this technique, two parts of the dialyzer are separated by a semi-permeable membrane which prevents the transfer of high molecular weight compounds and plasma proteins from one part of the dialyzer to the other but allows for the transfer of the low molecular substances between the device parts. In turn, UF has been introduced as an alternative to the equilibrium dialysis. This method is considered to be similar except that the centrifugal force is used to transfer low molecular weight analytes across the membrane.

As presented in Table 1, the determined degree of CMC binding in the case of using the ED and UF does not exceed the level of 81%. This indicates relatively strong binding of CMC to the plasma proteins; however, a significant amount of free drug is still present in the samples. As mentioned earlier, only the free fraction of the analyte has a therapeutic effect and thus the high content of the unbound form of the substance can cause its strong side effects shortly after its ingestion. This is exactly what takes place when CMC is introduced into the body. The literature data indicate that the strongest effect of CMC occurs just a few minutes after its consumption and lasts for several hours [4], as opposed to hydrophobic cannabinoids whose degree of binding to the plasma proteins is at the level reaching 99%.

Plasma or blood plasma is the liquid part of blood that is classified as an extracellular fluid. This is an aqueous solution of proteins, electrolyte substances and various organic and inorganic compounds. Blood plasma contains about 700 types of proteins. HSA is the most abundant plasma protein which, among its many functions, is responsible for the transport of exogenous and endogenous compounds. The exogenous compounds encompasses medicinal drugs and drugs of abuse. Transport properties of HSA are especially important for highly hydrophobic compounds as such substances exhibit a low solubility in the polar media like blood. However, binding of less hydrophobic substances also takes place [73, 74], thus affecting their pharmacodynamic and pharmacokinetic properties. In the case of 3-CMC and 4-CMC, the model studies employing NMR show their binding with HSA. This supports the results of protein-binding determination using separation techniques, given the importance of HSA for the drug transport in blood or plasma. The STD spectra and the determined epitope-binding maps show that all proton groups of the investigated CMCs interact with the HSA residues. Thus, it can be concluded that the whole 3-CMC and 4-CMC molecules are embedded in the binding sites of HSA. The most significant STD_0_ values were observed for the aromatic protons. As the WaterLOGSY spectra also show the most pronounced signals for the aromatic protons, i.e., the signal inversion, it can be seen that the results obtained from both NMR techniques correlate well and indicate that the aromatic ring plays a crucial role in binding of CMCs with HSA.

The extensive binding capacity of HSA is mainly due to the presence of two binding centers:  Sudlow Site I and  Sudlow Site II. 3-CMC and 4-CMC bind with both  Sudlow sites. It is a well-known fact that in the case of human serum albumin, the ligand binds first to its primary binding site and the remaining ligand molecules can interact with the secondary center [75].  Sudlow site II is the main binding center for 3-CMC and 4-CMC and  Sudlow site I acts as the secondary binding site. Such results are consistent with the paper by Patel et al. [48] in which interactions of mephedrone, i.e., 4-MMC, with the BSA were examined using several techniques and molecular docking simulations indicating that mephedrone binds in subdomain IIIA of the BSA. The ability of CMCs to bind to both Sudlow sites can be considered as an important factor influencing their transport throughout the organism. As the human blood contains numerous compounds that bind to HSA, the competition for the HSA binding sites between 3-CMC or 4-CMC with the other compounds is likely to occur. The results from the competition studies with warfarin and diazepam show that binding of CMCs to HSA still occurs, even in the presence of a compound that competes for a particular binding center.

The molecular simulation results correlate well with the literature data as the obtained docking positions of 3-CMC and 4-CMC within  Sudlow Sites are consistent with the typical dispositions of ligands in these cavities [71, 73, 74]. The determined dissociation constants, being at the micromolar level, are consistent with the obtained binding degrees by the equilibrium techniques, i.e., the values close to 80%, whereas the higher binding degrees would result in the dissociation constants at the low micromolar level as for the warfarin and diazepam [52, 53]. Moreover, binding energies calculated on the basis of molecular docking, where higher binding energies are obtained for the poses in Site II, confirm binding preferences for the  Sudlow Sites of HSA determined using the NMR spectroscopic techniques. Considering the results from the molecular dynamics, in which the obtained complexes of HSA with R-3-CMC were fully relaxed and equilibrated, it can be seen that the interactions of R-3-CMC with the docking sites residues are still present for these complexes in the equilibrium state, thus confirming the validity of the determined binding complexes.

Influence of the positional and optical isomerism of compounds on their pharmacokinetics and pharmacodynamics is of major concern. This also applies to the novel drugs of abuse [76, 77]. Positional isomerism of CMCs does not have significant influence on the binding degree with HSA as can be seen from the UF and ED experiments. This suggest that differences in docking parameters of 3- and 4-CMC binding with HSA observed using molecular docking can be considered as too small to affect the protein-binding degrees of CMC isomers. In the case of optical isomerism, it can be seen that differences of binding energies among the enantiomers observed for 3-CMC enantiomers are higher than the variations in binding of 4-CMC enantiomers. The observed variations in binding energies between the enantiomers do not exceed differences occurring between positional isomers. Although the binding of individual enantiomers of 3- and 4-CMC was not examined using experimental techniques, it can be assumed that, as in the case of positional isomers, the protein-binding degrees of 3- or 4-CMC enantiomers are similar.

## Conclusions

Although many studies describe pharmacological and toxicological properties of synthetic cathinones (SC), the data considering SCs binding in plasma are scarce. The equilibrium dialysis and ultrafiltration have been employed in order to determine the relationship between the fraction of free CMC and its total concentration. As results from the presented data, the degree of binding of 3- and 4-CMC is at a similar level of approx. 80% (79.1% and 79.3% for 3- and 4-CMC for the equilibrium dialysis and 80.2% and 80.5% for 3- and 4-CMC for ultrafiltration). This indicates a relatively strong binding of CMC to the plasma proteins, however, a significant amount of free drug is still present in the samples.

Model studies employing the NMR spectroscopic methods, i.e., STD and WaterLOGSY indicate that both isomers of CMC bind to HSA. The epitope maps for 3- and 4-CMC binding, in which the most pronounced contacts with the HSA residues were observed for aromatic moieties of CMC, were determined. Moreover, as can be seen from the competition STD experiments  Sudlow site II is a main binding center for 3- and 4-CMC and  Sudlow site I acts as a secondary binding site. For further investigations of the CMCs binding nature in  Sudlow sites I and II of HSA the molecular docking and molecular dynamic simulations were employed.

It is worth noting here that the novel psychoactive substances typically encompass a wide range of geometrical and optical isomers. Such structural variations can influence the biological/toxicological properties of illicit drugs. The influence of positional and optical isomerism of CMCs can be observed only at the atomic level using molecular simulations and model studies employing NMR spectroscopic techniques, but does not affect the overall drug binding, i.e., the plasma proteins biding degree.

To our knowledge, this is the first report presenting the comprehensive experimental and theoretical characterization of 3- and 4-CMC binding with the plasma proteins.

### Supplementary Information

Below is the link to the electronic supplementary material.

Fig. S1 1H NMR spectra for (a) 3-CMC (800 µM) with HSA (20 µM) and (b) 4-CMC (800 µM) with HSA (20 µM) obtained using the water suppression technique. Supplementary file1 (TIF 867 KB)

Fig. S2 Optimal docking poses of (a) R-3-CMC, (b) S-3-CMC, (c) R-4-CMC and (d) S-4-CMC in  Sudlow site II of HSA. Hydrogen bonds are represented by dashed lines. Supplementary file2 (TIF 7401 KB)

Fig. S3 Optimal docking poses of (a) R-3-CMC, (b) S-3-CMC, (c) R-4-CMC and (d) S-4-CMC in  Sudlow site I of HSA. Hydrogen bonds are represented by dashed lines. Supplementary file3 (TIF 5595 KB)

Fig. S4 Snapshots of (a) R-3-CMC embedded into  Sudlow site II and (b) R-3-CMC embedded into  Sudlow site I obtained from MD simulations. Supplementary file4 (TIF 3221 KB)

Fig. S5 Evolution of the distances between: R-3-CMC amine nitrogen atom and TYR 411 phenolic oxygen atom (blue line), R-3-CMC carbonyl oxygen atom and TYR 411 oxygen atom (red line), R-3-CMC amine nitrogen atom and ASN 391 amide carbonyl oxygen atom (green line) obtained from MD simulations of R-3-CMC in  Sudlow site II. Supplementary file5 (TIF 4993 KB)

Fig. S6 Evolution of the distance between: R-3-CMC amine nitrogen atom and GLN 196 amide carbonyl oxygen atom (blue line) obtained from MD simulations of R-3-CMC in  Sudlow site I. Supplementary file6 (TIF 2531 KB)

Supplementary file7 (DOCX 17 KB)

Supplementary file8 (DOCX 14 KB)

## Data Availability

All data generated or analyzed during this study are included in this article.
