# Supplementary figures and images for: Determination of 3- and 4-chloromethcathinone interactions with plasma proteins: study involving analytical and theoretical methods
Source: Forensic Toxicol. 2023 Dec 18;42(2):111–24. doi: 10.1007/s11419-023-00677-7 (PMC11269353; doi:10.1007/s11419-023-00677-7)

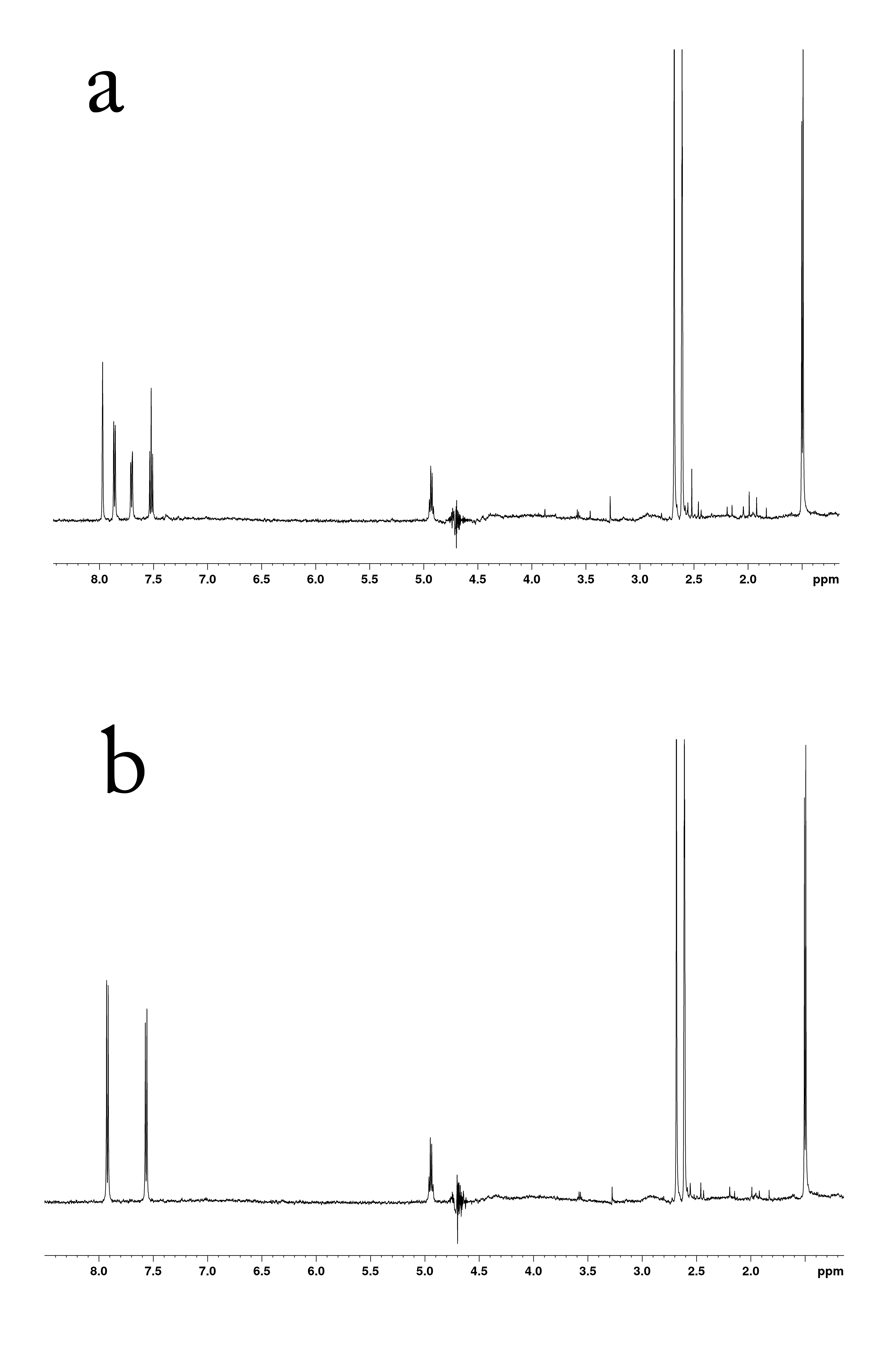

Supplement: Supplementary file 1 — Fig. S1 1H NMR spectra for (a) 3-CMC (800 µM) with HSA (20 µM) and (b) 4-CMC (800 µM) with HSA (20 µM) obtained using the water suppression technique. Supplementary file1 (TIF 867 KB) [file 11419_2023_677_MOESM1_ESM.tif]

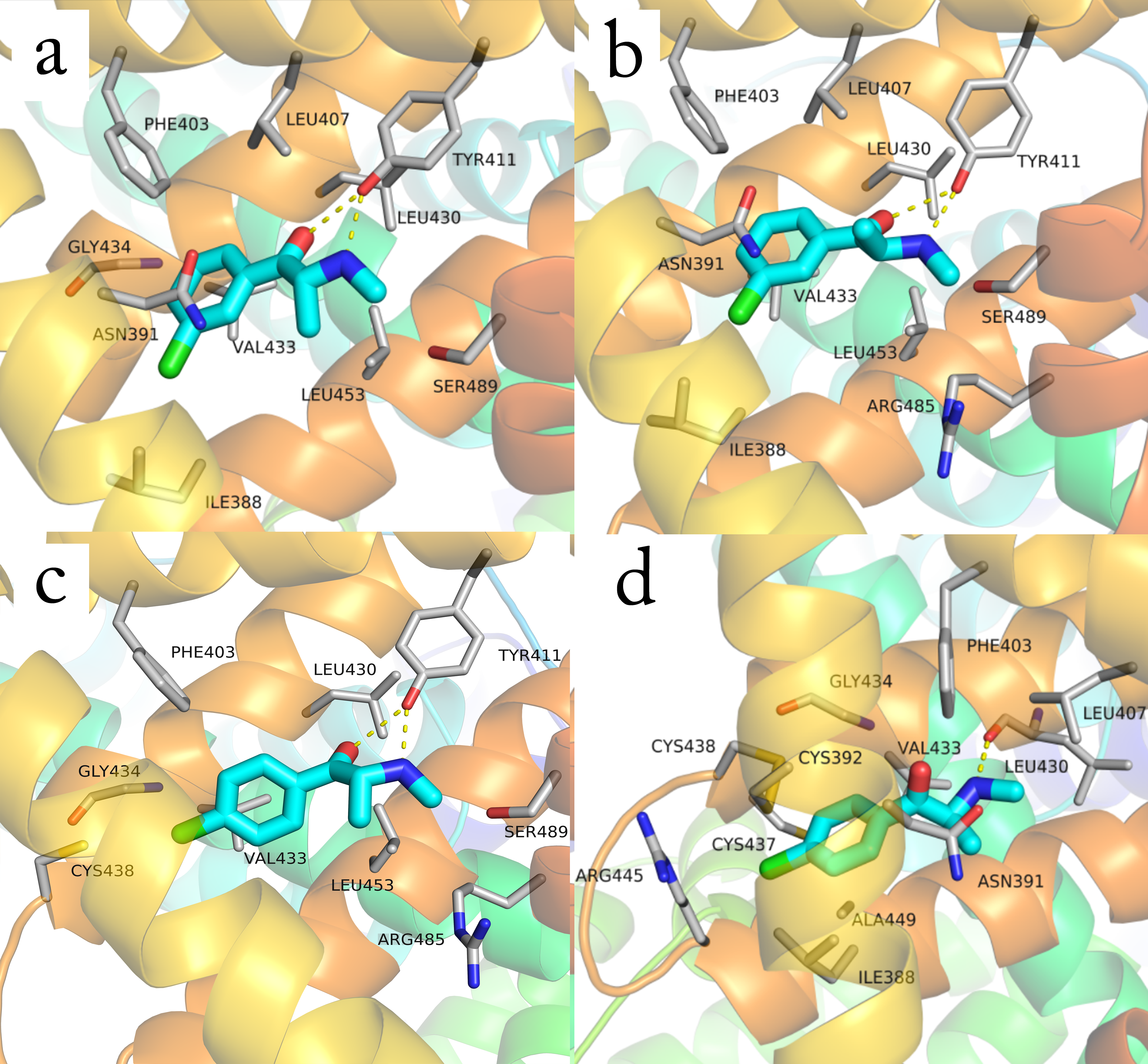

Supplement: Supplementary file 2 — Fig. S2 Optimal docking poses of (a) R-3-CMC, (b) S-3-CMC, (c) R-4-CMC and (d) S-4-CMC in Sudlow site II of HSA. Hydrogen bonds are represented by dashed lines. Supplementary file2 (TIF 7401 KB) [file 11419_2023_677_MOESM2_ESM.tif]

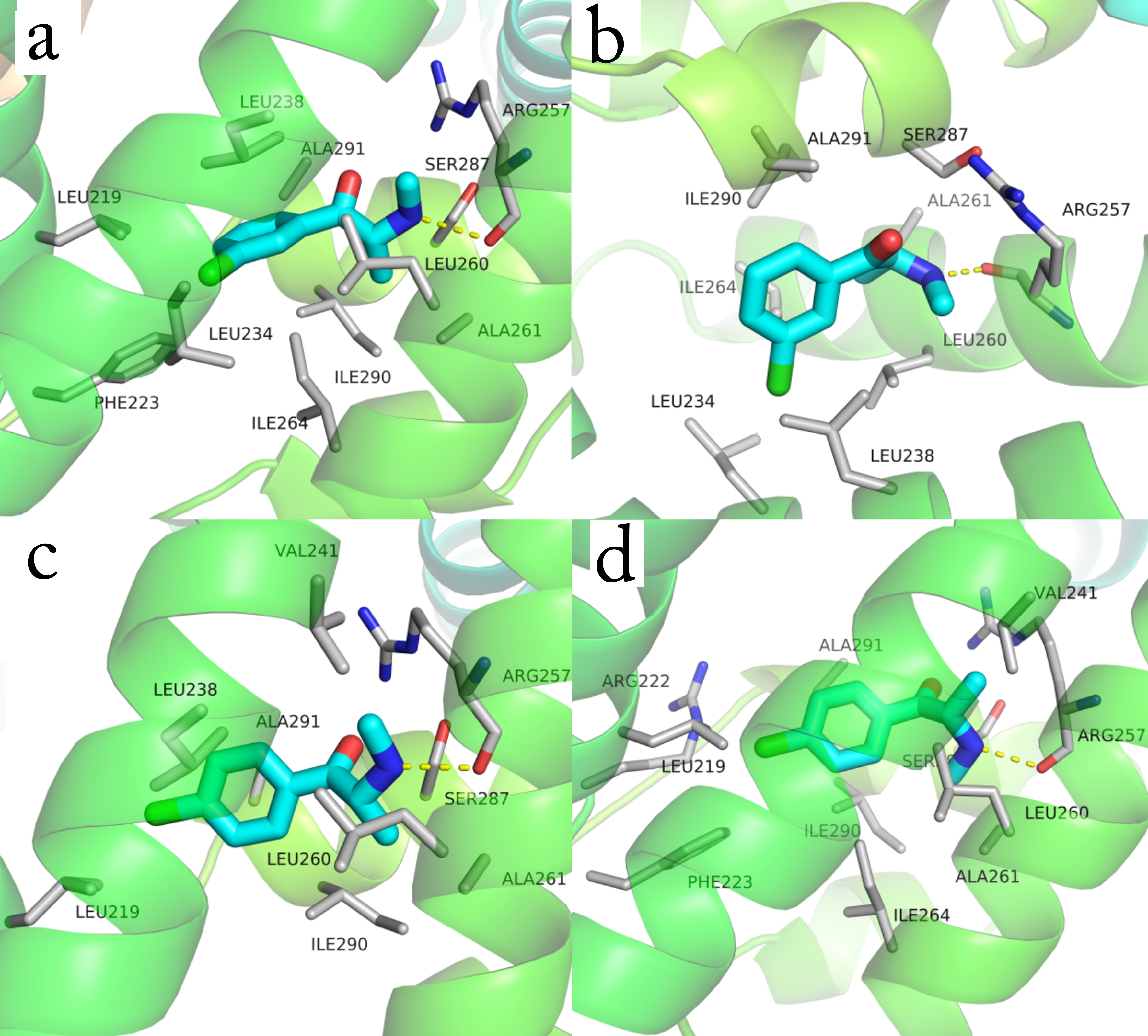

Supplement: Supplementary file 3 — Fig. S3 Optimal docking poses of (a) R-3-CMC, (b) S-3-CMC, (c) R-4-CMC and (d) S-4-CMC in Sudlow site I of HSA. Hydrogen bonds are represented by dashed lines. Supplementary file3 (TIF 5595 KB) [file 11419_2023_677_MOESM3_ESM.tif]

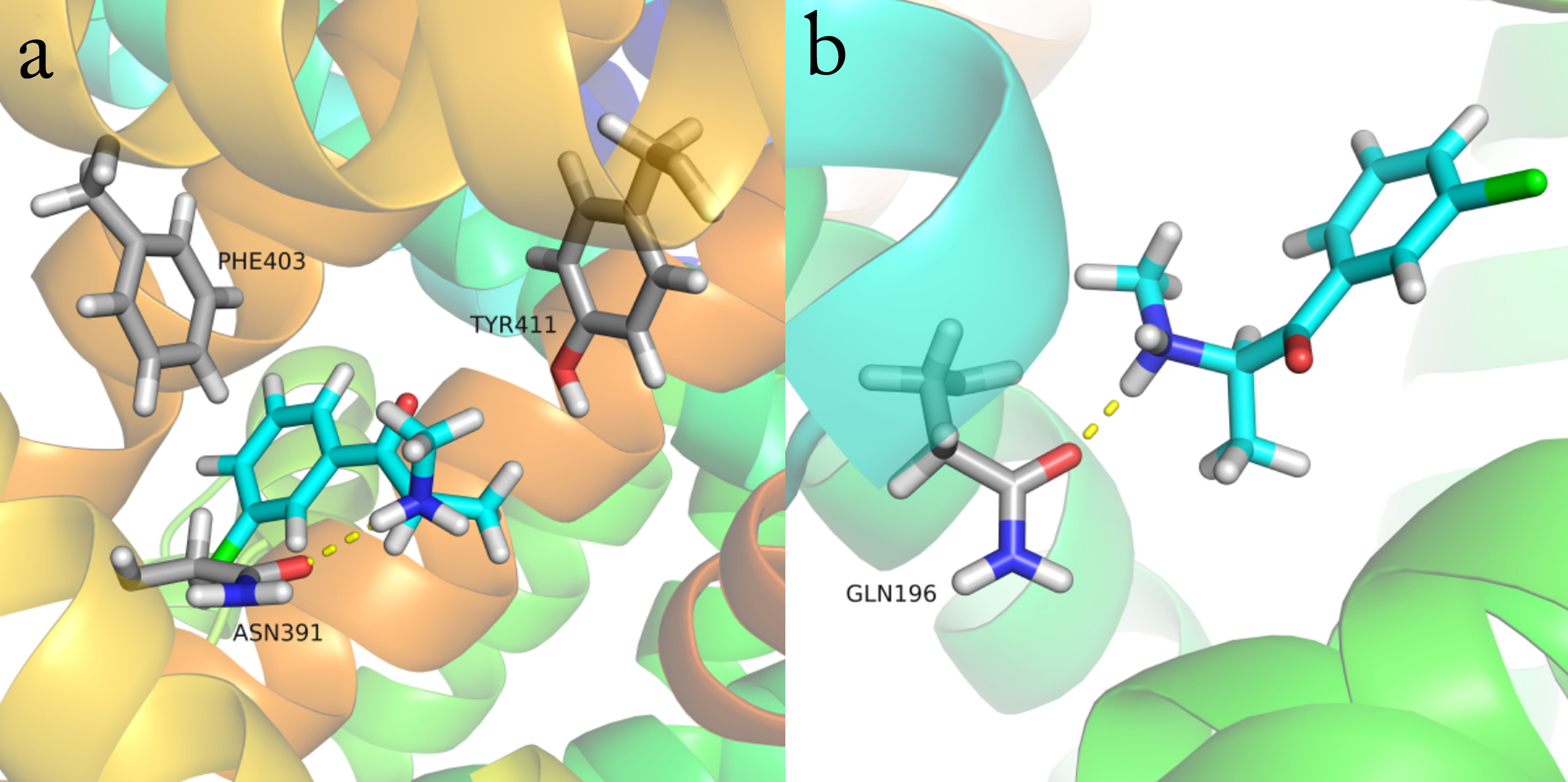

Supplement: Supplementary file 4 — Fig. S4 Snapshots of (a) R-3-CMC embedded into Sudlow site II and (b) R-3-CMC embedded into Sudlow site I obtained from MD simulations. Supplementary file4 (TIF 3221 KB) [file 11419_2023_677_MOESM4_ESM.tif]

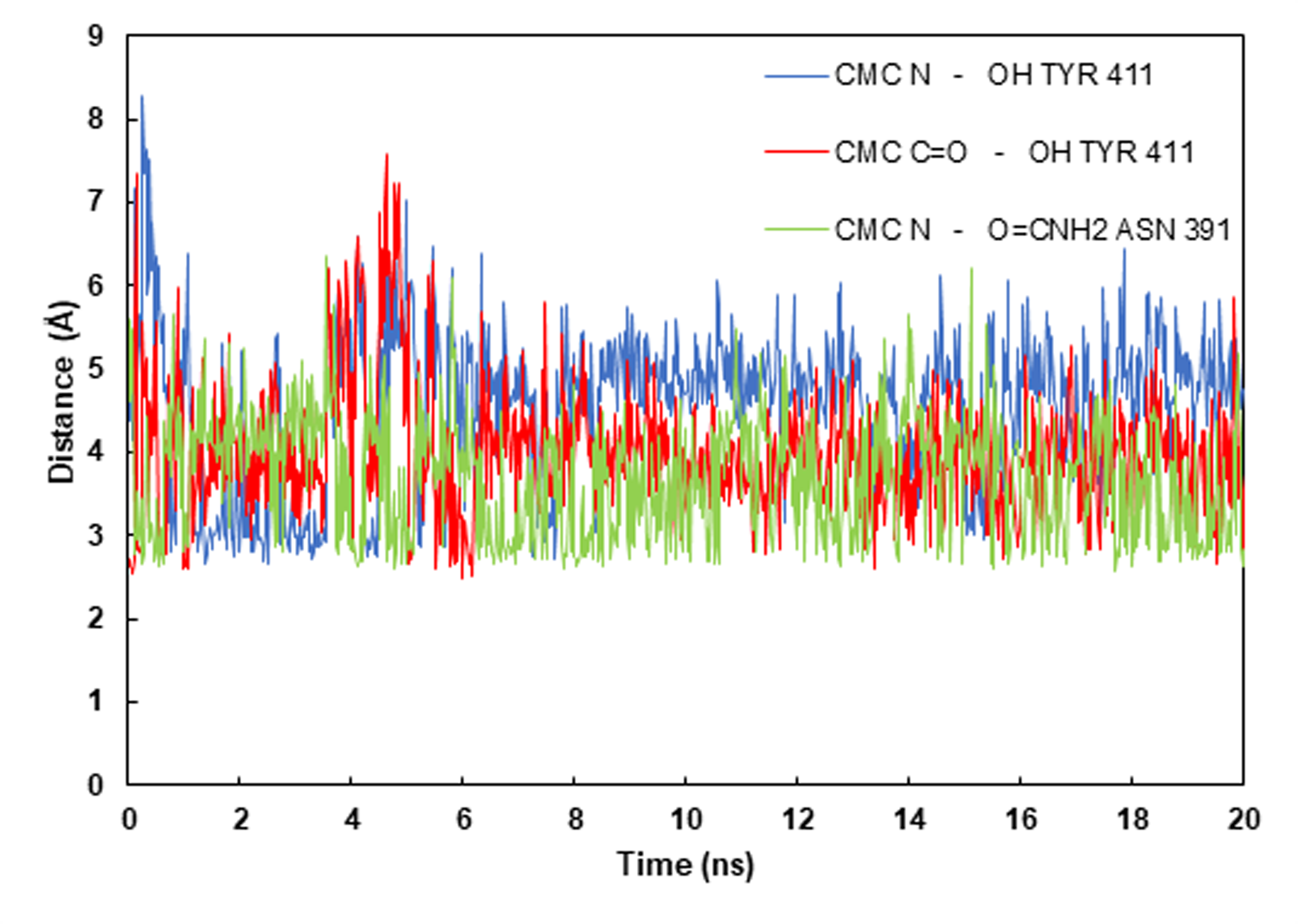

Supplement: Supplementary file 5 — Fig. S5 Evolution of the distances between: R-3-CMC amine nitrogen atom and TYR 411 phenolic oxygen atom (blue line), R-3-CMC carbonyl oxygen atom and TYR 411 oxygen atom (red line), R-3-CMC amine nitrogen atom and ASN 391 amide carbonyl oxygen atom (green line) obtained from MD simulations of R-3-CMC in Sudlow site II. Supplementary file5 (TIF 4993 KB) [file 11419_2023_677_MOESM5_ESM.tif]

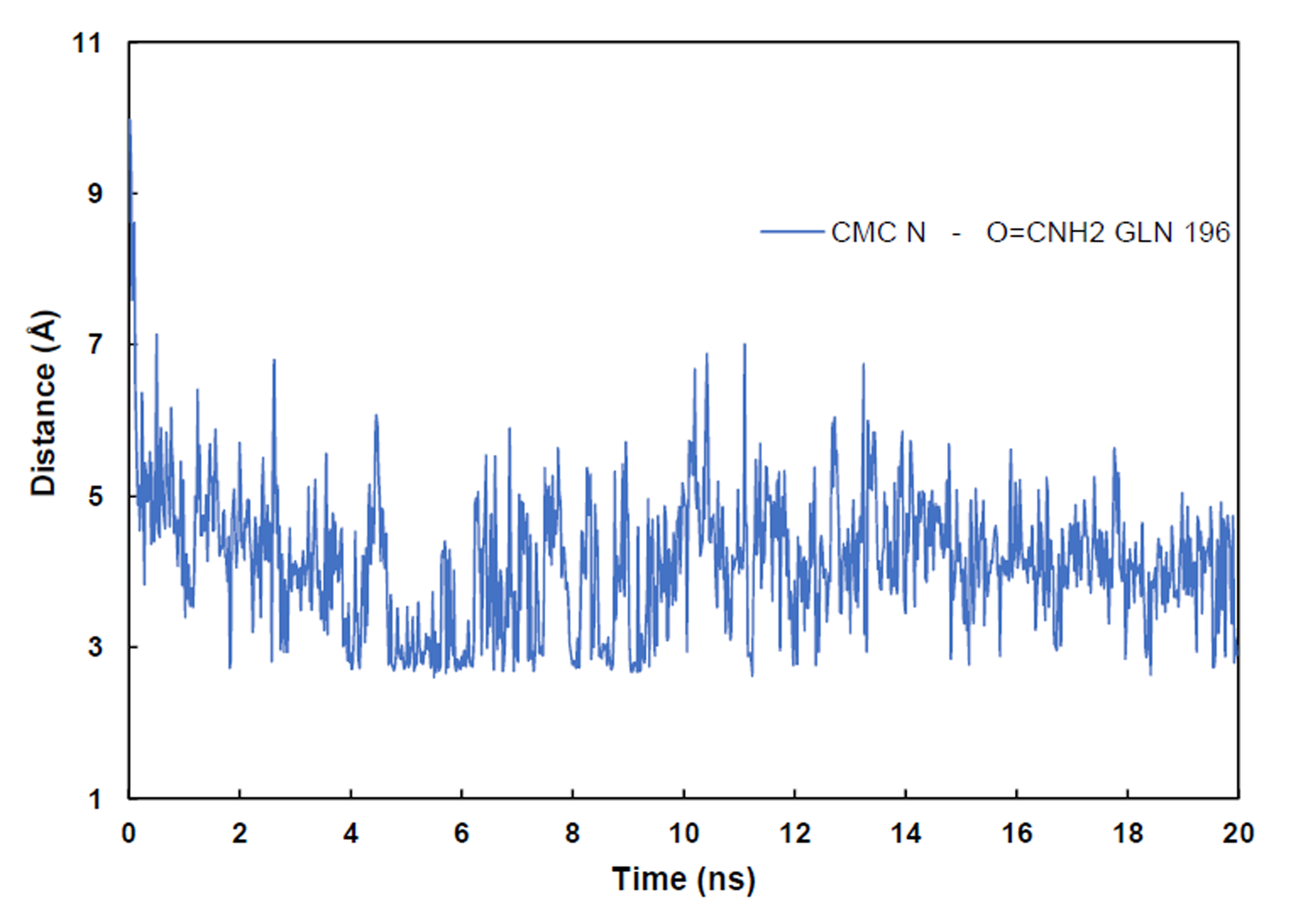

Supplement: Supplementary file 6 — Fig. S6 Evolution of the distance between: R-3-CMC amine nitrogen atom and GLN 196 amide carbonyl oxygen atom (blue line) obtained from MD simulations of R-3-CMC in Sudlow site I. Supplementary file6 (TIF 2531 KB) [file 11419_2023_677_MOESM6_ESM.tif]
