# Supplementary material for: Determination of 3- and 4-chloromethcathinone interactions with plasma proteins: study involving analytical and theoretical methods
Source: Forensic Toxicol. 2023 Dec 18;42(2):111–24. doi: 10.1007/s11419-023-00677-7 (PMC11269353; doi:10.1007/s11419-023-00677-7)
Supplement: Supplementary file 7 — Supplementary file7 (DOCX 17 KB) [file 11419_2023_677_MOESM7_ESM.docx]

Table S1. Fitting parameters for 3-CMC and 4-CMC build-up curves~~.~~ obtained from series of STD spectra with increasing saturation times.

| ^1^H δ [ppm] | STD_MAX_ | k _sat_ (s^-1^) | STD_0_ | Normalized STD_0_ |
| --- | --- | --- | --- | --- |
| 3-CMC | | | | |
| 7.99 | 23.40 | 0.10 | 2.34 | 81% |
| 7.87 | 6.55 | 0.35 | 2.29 | 79% |
| 7.71 | 10.33 | 0.28 | 2.89 | 100% |
| 7.53 | 6.07 | 0.47 | 2.85 | 99% |
| 4.90 | 0.50 | 1.33 | 0.67 | 23% |
| 2.69 | 3.02 | 0.36 | 1.09 | 38% |
| 1.50 | 2.50 | 0.38 | 0.95 | 33% |
| 4-CMC | | | | |
| 7.93 | 8.67 | 0.29 | 2.51 | 79% |
| 7.57 | 10.61 | 0.30 | 3.18 | 100% |
| 4.96 | 2.37 | 0.45 | 1.07 | 34% |
| 2.69 | 3.94 | 0.37 | 1.46 | 46% |
| 1.51 | 2.84 | 0.46 | 1.31 | 41% |
