# Supplementary material for: Determination of 3- and 4-chloromethcathinone interactions with plasma proteins: study involving analytical and theoretical methods
Source: Forensic Toxicol. 2023 Dec 18;42(2):111–24. doi: 10.1007/s11419-023-00677-7 (PMC11269353; doi:10.1007/s11419-023-00677-7)
Supplement: Supplementary file 8 — Supplementary file8 (DOCX 14 KB) [file 11419_2023_677_MOESM8_ESM.docx]

Table S2. Binding parameters of 3-CMC and 4-CMC docking modes in HSA Sudlov sites.

|  | Site I | | Site II | |
| --- | --- | --- | --- | --- |
|  | binding energy [kcal/mol] | K_D_ [µM] | binding energy [kcal/mol] | K_D_ [µM] |
| R-3-CMC | -5.34 | 121.93 | -6.67 | 12.99 |
| S-3-CMC | -5.69 | 67.18 | -6.32 | 23.38 |
| R-4-CMC | -5.07 | 192.38 | -6.43 | 19.43 |
| S-4-CMC | -5.18 | 160.47 | -6.47 | 17.94 |
